# Cooking frequency in Germany

**DOI:** 10.17886/RKI-GBE-2016-043

**Published:** 2016-12-14

**Authors:** Anja Borrmann, Gert B.M. Mensink

**Affiliations:** Robert Koch Institute, Department for Epidemiology and Health Monitoring, Berlin, Germany

**Keywords:** NUTRITION, COOKING HABITS, HEALTH SURVEY, DEGS1, GERMANY

## Abstract

The growing range of ready-to-cook and ready-to-eat foods (convenience foods) and opportunities to eat outside of the home is tending to reduce the number of fresh meals that people prepare themselves. However, people continue to place great importance on preparing their own meals from fresh foods, as this provides them with greater influence over the quality and composition of the food they eat. The German Health Interview and Examination Survey for Adults (DEGS1, 2008–2011) observed that 50.8% of adults aged between 18 and 79 (61.4% of all women and 40.2% of all men) prepare their meals daily or almost daily from fresh food. Moreover, women report less often that they never cook (2.9%) than men (16.1%) and older participants report far more often to cook daily or almost daily than younger participants. Finally, among both genders is a low level of employment associated with an increased cooking frequency.

## Introduction

A balanced diet is important to maintain and improve health. Numerous studies show that diet plays a significant role in the development of many ‘diseases of affluence’ such as obesity and diabetes [1]. Cooking habits may have a significant impact on people’s diets [2]. Therefore, an understanding of cooking habits in Germany could help develop approaches that would improve healthy nutrition promotion. Changes in society, to people’s living conditions and developments in the food sector are bringing people to eat more often convenience products and foods outside of their home [3, 4]. In today’s society, people expect food to be constantly available and easily accessible [5]. However, such products significantly reduce the influence that consumers have over the quality and composition of the foods they eat [6]. Moreover, many highly processed convenience foods have a relatively low protein contents and high levels of carbohydrates and fats [7].

The preparation of (warm) meals not only helps people understand more about the composition of foods, but also about the ingredients and the implications these have on their health. People who cook more often tend to engage more with issues related to food and preparing them. Consequently, studies demonstrate a positive correlation between a person’s knowledge about food and the quality of their diet: the more someone knows about healthy eating, the more often they eat nutritionally preferable foods such as fruit and vegetables [8–10]. Nevertheless, putting dietary recommendations into practice strongly depends on a person’s living conditions and their economic situation.

## Indicator

The German Health Interview and Examination Survey for Adults (DEGS1), which was conducted by the Robert Koch Institute between 2008 and 2011, gathered data on the frequency of cooking in people aged 18 to 79 [11]. The study participants were asked to complete a self-administered food frequency questionnaire that included the following question: ‘How many times per week do you prepare a hot meal (lunch or supper) yourself using basic ingredients/fresh foods?’ The possible answers were grouped into three categories: ‘every day/almost every day’, ‘1 to 4 times a week’, and ‘never’. A total of 6,956 participants provided information about how often they cook, and this data was statistically analysed. The following describes cooking frequencies according to gender, age, education and employment status.

The information on educational status was categorised using the CASMIN index (Comparative Analysis of Social Mobility in Industrial Nations). This index reflects the various levels present within the education system and takes into account the differences between vocational training and general educational pathways [12]. The participants’ employment status at the time of the survey was grouped into four categories: ‘full-time employment or training’, ‘part-time or marginal employment’, ‘unemployed or rarely in employment’, and ‘retired/pensioner’.

## Reflection of the results

According to DEGS1, 50.8% of adults in Germany prepare their warm meals every day or almost every day from fresh, basic ingredients (Table 1). The proportion of women who report this is significantly higher (61.4%) than the proportion of men (40.2%). In contrast, men report significantly more often that they never cook (16.1%) than women (2.9%). Still, 35.6% of women and 43.7% of men prepare their meals at least 1 to 4 times a week. Similar differences in cooking behaviour between men and women were observed in a study conducted on behalf of the Federal Ministry of Food and Agriculture [13]. One reason for these gender-specific differences might be that women place greater emphasis on a healthy diet, and are more likely to be responsible for caring for the family [13, 14].

Alongside gender-specific differences, DEGS1 also demonstrates age-related differences in terms of cooking frequency. Participants in the oldest age group (65–79 years) cook the most often, with 81.9% of women and 60.6% of men in this age group cooking every day or almost every day. This confirms the widespread hypothesis that dietary and cooking behaviour differs considerably between the generations [15, 16]. The age-specific differences in cooking habits could be explained by the fact that older people tend to adhere to more traditional practices and probably also are more health conscious and consequently care more about their diet [10]. Moreover, most people in this age group no longer have to work, which means that they have more time to cook their own food. In contrast, today’s younger generation has grown up in a period offering a vast availability of diverse industrially processed convenience foods, and catering services [3, 17]. DEGS1 demonstrates that young women and men consume fast food particularly often compared with older people [18].

**Fig. 1:**
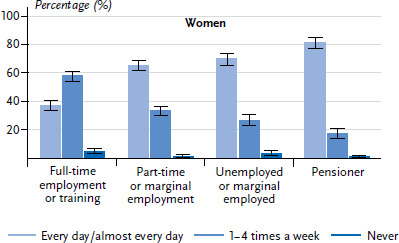
Preparation of meals by 18- to 79-year-old women according to employment status (n=3,600) Source: DEGS1 (2008–2011)

**Fig. 2:**
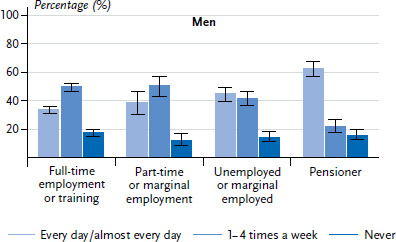
Preparation of meals by 18- to 79-year-old men according to employment status (n=3,303) Source: DEGS1 (2008–2011)

A low level of employment is also linked to a higher frequency of cooking (Figures 1 and 2). People who are unemployed, in marginal employment and pensioners cook significantly more often every day or almost every day than those who are in full-time employment. This could be because the majority of people in full-time employment have less time available during the day to cook for themselves. This could be reflected in a propensity to eat outside of the home far more often and in a preference for cooking on weekends [5]. Among all age groups, there is a tendency, for both women and men with a low education level to cook more often every day or almost every day then those with a medium or higher level of education. However, these differences are not statistically significant (Table 2).

Further targeted studies are needed to accurately assess cooking habits and their impact on dietary behaviour and health. The basic ingredients used and the way in which a meal is prepared may be more important for health than mere cooking frequency [19]. Nevertheless, consumers who do cook have more influence over the type of food they eat and may find it easier to implement health recommendations, such as reducing salt levels. Moreover, people who regularly cook their own meals are more knowledgeable when it comes to handling food and often have better cooking skills. Another study observed that cooking abilities, particularly among people with lower socio-economic statuses, played a significant role in enabling these individuals to maintain a healthy diet [2]. Measures aimed at increasing cooking frequency, therefore, could presumably play a role in improving the population’s dietary habits.

## Key statements

- A total of 50.8% of adults prepare their meals from fresh food every day or almost every day.
- The proportion of women who cook every day or almost every day (61.4%) is much higher than the proportion of men who do so (40.2%).
- Older women and men are proportionately more likely to cook every day or almost every day than younger people.
- A higher proportion of pensioners and people who are only in marginal employment cook every day or almost every day than those in full-time employment.

## Figures and Tables

**Table 1:** Cooking frequency among 18- to 79-year-olds according to gender and age Source: DEGS1 (2008–2011)

Age					
	18 – 29 years	30 – 44 years	45 – 64 years	65 – 79 years	Total
	% (95% Cl)	% (95% Cl)	% (95% Cl)	% (95% Cl)	% (95% Cl)
**Women**	**n = 535**	**n = 743**	**n = 1,455**	**n = 903**	**n = 3,636**
Every day/almost every day	39.9 (34.6 – 45.5)	57.8 (53.5 – 61.9)	63.0 (59.8 – 66.1)	81.9 (77.7 – 85.5)	61.4 (59.1 – 63.8)
1 – 4 times a week	53.0 (47.7 – 58.1)	39.7 (35.8 – 43.7)	35.0 (32.0 – 38.2)	16.7 (13.2 – 20.8)	35.6 (33.4 – 37.9)
Never	7.1 (4.6 – 10.9)	2.6 (1.4 – 4.5)	1.9 (1.2 – 3.1)	1.4 (0.8 – 2.6)	2.9 (2.2 – 3.8)
**Men**	**n = 512**	**n = 663**	**n = 1,255**	**n = 890**	**n = 3,320**
Every day/almost every day	31.0 (26.1 – 36.5)	34.7 (29.6 – 40.2)	39.0 (35.4 – 42.7)	60.6 (55.5 – 65.4)	40.2 (37.8 – 42.7)
1 – 4 times a week	52.5 (47.4 – 57.6)	51.2 (46.2 – 56.2)	44.2 (40.4 – 48.0)	22.3 (18.6 – 26.5)	43.7 (41.7 – 45.7)
Never	16.4 (13.1 – 20.4)	14.1 (10.7 – 18.3)	16.8 (14.6 – 19.3)	17.1 (13.9 – 21.0)	16.1 (14.3 – 18.0)

CI = confidence interval

**Table 2:** Cooking frequency among 18- to 79-year-olds according to gender, age and educational level (n=3,614 women, n=3,302 men) Source: DEGS1 (2008–2011)

Educational level				
		Low	Medium	High
		% (95% Cl)	% (95% Cl)	% (95% Cl)
**Women**		**n = 1,175**	**n = 1,878**	**n = 561**
**Age**				
18 – 29 years	Every day/almost every day	47.8 (34.3 – 61.6)	38.8 (31.9 – 46.1)	38.3 (23.4 – 55.8)
	1 – 4 times a week	41.5 (28.0 – 56.4)	54.9 (48.0 – 61.7)	54.1 (39.2 – 68.4)
	Never	10.7 (5.1 – 21.2)	6.3 (4.1 – 9.6)	7.6 (2.1 – 23.6)
30 – 44 years	Every day/almost every day	69.7 (60.8 – 77.3)	55.4 (49.2 – 61.5)	53.4 (43.8 – 62.8)
	1 – 4 times a week	28.2 (20.6 – 37.4)	42.7 (36.9 – 48.7)	43.6 (35.1 – 52.4)
	Never	2.1 (0.6 – 7.2)	1.9 (0.9 – 3.8)	3.0 (0.8 – 10.4)
45 – 64 years	Every day/almost every day	69.7 (64.3 – 74.6)	59.1 (54.9 – 63.1)	58.5 (50.5 – 66.1)
	1 – 4 times a week	28.3 (23.6 – 33.6)	38.9 (34.9 – 43.0)	40.6 (33.1 – 48.6)
	Never	2.0 (1.0 – 4.0)	2.1 (1.0 – 4.3)	0.9 (0.3 – 2.4)
65 – 79 years	Every day/almost every day	83.1 (78.4 – 87.0)	80.8 (73.2 – 86.6)	76.6 (62.0 – 86.8)
	1 – 4 times a week	15.1 (11.5 – 19.6)	18.3 (12.6 – 26.0)	23.4 (13.2 – 38.0)
	Never	1.7 (0.9 – 3.5)	0.9 (0.2 – 3.5)	-
**Men**		**n = 1,067**	**n = 1,485**	**n = 750**
**Age**				
18 – 29 years	Every day/almost every day	33.5 (23.2 – 45.6)	32.3 (26.5 – 38.7)	16.5 (8.2 – 30.5)
	1 – 4 times a week	43.0 (31.8 – 55.0)	51.9 (45.8 – 58.1)	74.9 (59.7 – 85.7)
	Never	23.5 (15.1 – 34.6)	15.7 (11.7 – 20.8)	8.6 (2.9 – 22.8)
30 – 44 years	Every day/almost every day	38.4 (27.5 – 50.6)	34.2 (28.2 – 40.7)	30.9 (22.6 – 40.7)
	1 – 4 times a week	45.4 (34.9 – 56.4)	53.9 (47.4 – 60.2)	51.0 (41.1 – 60.7)
	Never	16.1 (9.7 – 25.7)	11.9 (8.4 – 16.7)	18.1 (12.5 – 25.5)
45 – 64 years	Every day/almost every day	41.1 (35.2 – 47.3)	37.7 (32.6 – 43.1)	37.9 (31.2 – 45.1)
	1 – 4 times a week	41.9 (35.9 – 48.2)	45.3 (39.8 – 50.9)	45.8 (39.3 – 52.5)
	Never	17.0 (13.2 – 21.5)	17.0 (13.9 – 20.7)	16.3 (11.6 – 22.4)
70 – 79 years	Every day/almost every day	60.9 (54.4 – 67.0)	61.3 (51.1 – 70.5)	58.9 (49.5 – 67.7)
	1 – 4 times a week	20.9 (16.1 – 26.8)	25.1 (16.9 – 35.5)	23.7 (17.0 – 32.1)
	Never	18.2 (13.9 – 23.3)	13.7 (8.5 – 21.3)	17.3 (10.9 – 26.4)

CI = confidence interval
